# An Adaptive Measure of Visuospatial Impairment in Dementia with Lewy Bodies

**DOI:** 10.1002/mdc3.13488

**Published:** 2022-06-13

**Authors:** Joseph R. Phillips, Elie Matar, Kaylena A. Ehgoetz Martens, Ahmed A. Moustafa, Glenda M. Halliday, Simon J.G. Lewis

**Affiliations:** ^1^ Faculty of Medicine and Health, Brain and Mind Centre and Central Clinical School University of Sydney Sydney New South Wales Australia; ^2^ School of Psychology & Marcs Institute for Brain and Behaviour Western Sydney University Sydney New South Wales Australia; ^3^ Dementia and Movement Disorders Laboratory, Brain and Mind Centre University of Sydney Sydney New South Wales Australia; ^4^ Department of Kinesiology, Faculty of Health University of Waterloo Waterloo Ontario Canada

**Keywords:** dementia with Lewy bodies (DLB), Parkinson's disease (PD), visuospatial impairment, attention, mental rotation

## Abstract

**Background:**

Dementia with Lewy bodies (DLB) is a common cause of dementia with poor prognosis and high hospitalization rates. DLB is frequently misdiagnosed, with clinical features that overlap significantly with other diseases including Parkinson's disease (PD). Clinical instruments that discriminate and track the progression of cognitive impairment in DLB are needed.

**Objectives:**

The current study was designed to assess the utility of a mental rotation (MR) task for assessing visuospatial impairments in early DLB.

**Methods:**

Accuracy of 22 DLB patients, 22 PD patients and 22 age‐matched healthy controls in the MR task were compared at comparing shapes with 0°, 45° and 90° rotations.

**Results:**

Healthy controls and PD patients performed at similar levels while the DLB group were significantly impaired. Further, impairment in the visuospatial and executive function measures correlated with MR poor outcomes.

**Conclusion:**

These findings support the MR task as an objective measure of visuospatial impairment with the ability to adjust difficulty to suit impairments in a DLB population. This would be a useful tool within clinical trials.

Dementia with Lewy Bodies (DLB) is the second most common cause of neurodegenerative dementia in the elderly, accounting for up to 5–20% of dementia cases.^1^, ^2^, ^3^, ^4^ This neurodegenerative disease is characterized by an aggressive decline that significantly impacts on everyday activities^5^ with a poor prognosis that frequently requires hospitalization or full‐time care.^6^ Whilst diagnostic criteria have been established to facilitate an earlier and more accurate diagnosis by recognizing four core features (parkinsonism, cognitive fluctuations, visual hallucinations and dream enactment),^7^ many patients remain undiagnosed or misdiagnosed.^1^, ^4^ The frequent presence of parkinsonism and REM Sleep behavior disorder (RBD) will usually help the clinician in differentiating DLB from non‐synucleinopathies. Conversely, even at the time of first diagnosis, around a third of Parkinson's Disease (PD) patients are known to have Mild Cognitive Impairment (MCI)^8^ and almost half of PD patients have RBD.^9^ Indeed, differentiating these two synucleinopathies from each other can prove challenging. Thus, there is an immediate need to find convenient approaches to assist in the recognition of such cases.

Whilst the cognitive decline observed in DLB may encompass a broad range of domains, the most significant impairments occur across executive^10^ and visuospatial function.^11^, ^12^ Indeed, it has been reported that even during the prodromal (classified as MCI with two of the core diagnostic features)^13^ and early stages of the disease, DLB patients have more visuospatial impairments than PD patients with a similar disease duration.^14^ Therefore, this differential impairment of visuoperceptive abilities may represent a suitable neuropsychometric target that could be utilized to differentiate early DLB cases from other conditions with similar initial clinical profiles, such as PD.

A range of tests investigating visuospatial ability have been employed in patients with DLB, but these results have lacked consistency. This may be due to the heterogeneity of the disease or the nature of other disease features such as cognitive fluctuations, which are common in DLB and would clearly impact upon the performance of many tasks. The Visual Object and Space Perception battery (VOSP) was originally designed to help differentiate DLB patients with a disease duration of at least 12 months from Alzheimer's disease (AD) patients.^15^ The task requires subjects to complete a series of trials designed to measure an individual's spatial and object perception.^16^ The VOSP consists of nine simple tasks that involve the identification of obscure shapes and silhouettes by matching numbers to locations.^17^ However, applying the VOSP to prodromal DLB patients has produced mixed results. Van de Beek and colleagues^18^ found more impairment in prodromal DLB patients compared to prodromal AD patients, however Kemp and colleagues^12^ were unable to separate prodromal DLB patients from either AD or healthy controls using the VOSP. This suggest that the VOSP may not be sensitive enough to discriminate between neurodegenerative conditions.

The Benton Line Orientation Judgment task is a 30‐item task that also measures visuospatial ability by having patients compare and match line orientations. There are few studies that have used this task to directly compare DLB with other synucleinopathies. A recent study found that DLB patients were more impaired than PDD patients.^19^ However, almost half of the DLB patients were unable to complete the task and thus it may prove unsuitable for general use.^19^ The Rey‐Osterrieth Complex Figure copy (ROCF) is another popular test to measure visuospatial abilities in healthy and clinical populations.^20^ There are mixed findings regarding ROCF ability to differentiate DLB from PDD patients with one study reporting more impairment in DLB than PDD^19^ while another reporting no difference between the DLB and PDD.^21^ This discrepancy in findings is likely due to DLB and PDD patients in the latter study having less cognitive impairment than the former.

Previously, the Mental Rotation task has been utilized in PD,^22^ AD,^23^, ^24^, ^25^ Huntington's disease,^26^ and traumatic brain injury^27^ to evaluate an individual's ability to internally visualize and manipulate three dimensional (3D) objects.^28^ Participants are presented pairs of shapes and are required to determine if the shapes are identical or mirror images of each other.^28^ One shape is rotated on its y‐axis, which then requires the participant to visualize and rotate the shape to determine if it is the same or a mirror image of its pair. The amount of rotation has been shown to be correlated with greater cognitive load and subsequently higher difficulty as the rotation approaches 180°.^29^ This feature of the Mental Rotation task allows the difficulty to be adjusted to be more accessible to patients with differing degrees of visuospatial impairment.

Variations of the Mental Rotation task have already been tested with non‐demented PD patients. Duncombe and colleagues^30^ tested PD patients rotating 2D stick figures on the z‐axis and found that they performed as well as healthy controls. Employing a similar paradigm but replacing the figures with faces, Adduri and Marotta^23^ found impairments in AD patients that worsened as the degree of rotation increased. However, the extent of dementia was not controlled for, and the impaired domains were not specified. To date, Mental Rotation tasks have not been utilized in DLB but would appear to hold some utility in assessing visuospatial performance.

The aim of the current study was to assess a novel version of the Mental Rotation task, which incorporated 3D shapes with varying degrees of rotation on the y‐axis, to detect differences across DLB and PD patients with matched disease durations, as well as age matched healthy controls. We predicted that performance by the two patient groups would be more impaired than in the healthy controls, and that the DLB group would have significantly poorer accuracy than the PD group. Furthermore, it was anticipated that performance across attentional and visuospatial domains would be correlated with performance on the Mental Rotation task, as opposed to memory deficits.

## Methods

### Participants

Groups were matched on age, years of education and within the patient groups, disease duration. This resulted in a sample of 22 healthy controls, 22 DLB patients and 22 PD patients. Participants were recruited through the ForeFront Parkinson's Disease Research Clinic (University of Sydney). Informed consent was provided by each patient and the study was approved by the Human Research Ethics Committee at Sydney University. All patients underwent neurological assessment including the Movement Disorder Society Unified Parkinson's Disease Rating Scale for motor features (MDS‐UPDRS section III) and a comprehensive neuropsychological battery for cognitive impairments. PD patients were diagnosed using the Movement Disorder Society diagnostic criteria,^31^ DLB patients were diagnosed using according to the fourth diagnostic consensus criteria.^7^ Healthy controls were recruited generally as spouses, caregivers, or close relatives and screened for underlying conditions via a clinical interview. Participants were excluded if they less than 6/12 corrected vision or were unable to perform the task, this resulted in the exclusion of no participants. Patients were tested whilst taking their normal medications, with 17 DLB patients taking cholinesterase inhibitors (AChEI) and six DLB patients taking dopaminergic medications.

### Procedure

#### Mental Rotation Task

**FIG. 1:**
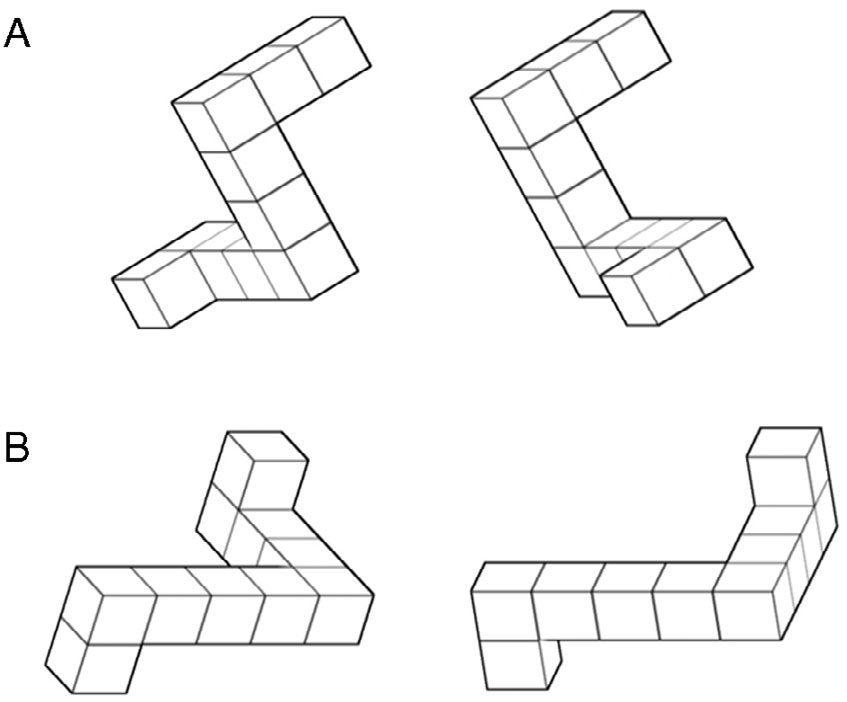
(**A**) A pair of mirror image shapes with 0° rotation. (**B**) A pair of identical shape with 45° rotation.

The Mental Rotation (MR) task was adapted from Shepard and Metzler^28^ using E‐Prime 2.10,^32^ which was presented on a 15” laptop. Pairs of shapes that were constructed from 10 cubes and contained two 90° bends (Fig. 1) were presented in front of the patient at a comfortable distance. Each shape was either an exact copy of its paired shape (Fig. 1A), or a mirror image (Fig. 1B). Participants were required to indicate if shapes within each pair were the same by pressing a key labeled “Same” or if they were a mirror image by pressing key labeled “Mirror.” Each pair was presented until a response was made or after 30 seconds the trial would timeout, which was scored as incorrect. The researcher would also prompt for an answer during trials to make sure the participant was still performing the task to avoid the potential confound of cognitive fluctuations. The initial Shepard and Metzler^28^ study presented the pairs with different ranges of rotation on the y‐axis from 0° to 180°. However, the current study only used three rotations 0°, 45° and 90°. These rotations proved simple enough for the cognitively impaired sample, whilst still providing enough variance for analysis. Before the test trials, participants were briefed on how to differentiate *same* pairs from *mirror* pairs and given practice examples to complete to ensure they knew how to correctly complete the task. Each rotation condition was presented 18 times in random order. The total number of trials was 54, with a break after 28 trials to minimize fatigue. The average run time was 20 minutes. The main measure of the MR task for the current study was mean accuracy and correct response time (RT) for each rotation condition. Changes in accuracy between difficulties 0°–45° and 0°–90° were also calculated to measure potential cost of increasing the rotation between the pairs by 45° or 90°. The minimum score was 50% as this would represent random chance.

### Clinical Measures

General level of cognitive impairment was measured using the Mini Mental State Examination (MMSE).^33^ Visual hallucinations were measured using the item 1.2 from MDS‐UPDRS, which rates the severity of hallucinations as part of a clinical interview. Severity is scored on a 5‐point Likert scale ranging from 0 (“*no hallucinations or psychotic behaviour*”) to 4 (“*Patient has delusions and paranoid behaviour*”). Hallucinations were also measured using the Psychosis and Hallucinations Questionnaire (PsycH‐Q). The PsycH‐Q consists of two main subscales, *Hallucinations and Psychosis* and *Hallucination Phenotypes*. The first subscale probes the severity of hallucinations (e.g. “*over the last month how often did you…*” “*feel like there is something lurking in the corner of your vision*” or “*see people or things that aren't there*”). Other modalities of hallucinations are also identified (i.e. auditory, tactile, olfactory and gustatory), along with features of psychosis (i.e. disordered thoughts or presence of delusion). The second subscale measures symptoms that commonly co‐occur with hallucinations (i.e. attention difficulties, vivid dreams and enactment of dreams). Frequency of hallucinatory behaviors and features are measure on a 5‐point Likert scale ranging from 0 (“*never*”) to 4 (“*daily*”). The PsycH‐Q has been validated in a non‐demented PD population.^34^, ^35^ Cognitive fluctuations were also measured using the One Day Fluctuation scale.^36^ This scale measures the prevalence of symptoms associated with fluctuations as observed by a caregiver. Symptoms included impaired attention, communication, drowsiness, disorganized thinking, consciousness, falls and fluctuations of impairment.^36^ Scores range from 0 (no fluctuations) to 21 (severe fluctuations).

### Neuropsychological Battery

Neuropsychological tests included the copy component of the Rey Complex Figure task (RCF),^20^ Trail‐making task parts A and B,^37^ digit span,^38^ Stroop test,^39^ Controlled Oral Word Association Test^40^ and the clock drawing component of the MoCA. Additionally, memory impairment was measured using the Digit span forward and Logical Memory task.^41^

### Statistics

Demographics were compared across the groups using Kruskal‐Wallis and Mann–Whitney U tests. Performance of the MR task was analyzed using a 3 (HC, PD, DLB) × 3 (0°, 45°, 90°) factorial, mixed repeated measures ANOVA. Due to deviations from normal distribution, potential correlations between MR performance and selected neuropsychological tests were analyzed using Spearman correlations. A receiver operator characteristic (ROC) curve was also calculated to determine the accuracy the MR task as at identifying DLB patients from PD and HC patients.

## Results

### Demographics

Demographic differences are listed in Table 1. All groups were matched on age, years of education and the two patient groups were matched on their disease duration. There were many more females in the HC group compared to the patient groups due to the higher incidence of DLB and PD in males and the use of spousal controls. Parkinson's disease patients were on a higher Dopamine Dose Equivalent (DDE) (M = 730, SD 210) than the DLB group (M = 152, SD 246). As anticipated, the groups differed on the MMSE score with DLB (M = 22.2, SD = 6.2), having the poorer performance than HC (M = 29.3, SD = 0.91) and PD (M = 28.7, SD = 1.36) groups. While no difference between MMSE scores for the HC and PD group.

**TABLE 1:** Clinical and demographic characteristics of patient populations means (SD)

	HC	PD	DLB
N	22	22	22
Age	73.6 (5.9)	71.2 (3.6)	74.3(6.0)
Gender (males)	9	16	20
Years of education	14.0 (3.4)	13.6 (3.3)	12.4(3.5)
Disease Duration	n/a	1.18 (0.85)	0.45(1.10)
MMSE	29.3 (0.91)	28.7 (1.36)	22.2(6.2)^a,b^
DDE	n/a	273 (210)	152(244)^a^
Hallucinators	n/a	3	15
RBD	n/a	12	21
MDS‐UPDRS III	n/a	21.2 (8.10)	32.8(14.4)

a = DLB ≠ PD, *P* < 0.01; b = DLB ≠ HC, *P* < 0.01.

Abbreviations: HC, Healthy controls; PD, Parkinson's disease; DLB, Dementia with Lewy bodies; MMSE, Mini Mental State Examination; DDE, Daily Dopamine Equivalent.

### Performance on Mental Rotation

Differences in accuracy between groups and across difficulty levels in the Mental Rotation task was analyzed using a mixed repeated measures ANOVA (Table 2). The assumption of sphericity was not met, and a Huynh‐Feldt correction was used. Additionally, homogeneity of variance was not met, so a Games‐Howell correction was used for pairwise comparisons. The interaction of task difficulty and diagnosis was not significant F(3.47, 109.29) = 0.77, *P* = 0.53, *η* ^2^ = 0.02. However, there was a significant effect of rotation F(1.74,109.29) = 52.82, *P* < 0.001, *P* ^2^ = 0.46. Pairwise comparisons using Sidak correction showed that participants had the most difficulty on the 90° (*P* < 0.001), followed by the 45° (*P* < 0.001) rotation difficulty. There was also an effect of diagnosis, F(2, 63) = 10.13, *P* < 0.001, *η* ^2^ = 0.24. Adjusting the alpha to 0.017 for pairwise comparisons, it was found that this effect was driven by the DLB group having a lower accuracy than the HC and PD groups (*P* < 0.001, *P* = 0.007, respectively). There was no significant difference between the HC and PD groups. No difference was found between DLB patients who were taking AChEI and DLB patients not on AChEIs for any of the MR difficulty levels. Changes in accuracy between 0° and 45° were not different between groups, F(2, 63) = 0.44, *P* = 0.65 *η* ^2^ = 0.01, nor were they between the 0° and 90° conditions F(2,63) = 0.25, *P* = 0.8, *η* ^2^ = 0.01. Receiver operator analysis revealed that total accuracy scores on the MR were 80% accurate at distinguishing DLB patients from HC and PD groups. Setting an accuracy threshold of 80% provides a sensitivity and specificity score of 86% and 47% respectively. Response time was also compared using a mixed measures ANOVA. There was a significant interaction between diagnosis and difficulty F(4,114) = 5.61, *P* < 0.001, η = 0.16. To analyze this interaction further, simple effects were measured using a repeated measures ANOVA for each individual patient group. The effect of difficulty was only significant in the HC group (F (2, 42) = 3.74, *P* < 0.001, *η* ^2^ = 0.4) as PD and DLB groups were not significant after correcting for multiple comparisons (*P* = 0.07 & *P* = 0.03, respectively). Using pairwise comparison with Sidak correction, the HC group were significantly quicker at responding for the 0° condition compared with the 45° condition.

**TABLE 2:** Mean (SD) accuracy (%) and RT (ms) of correct trials for mental rotation task and change of accuracy and RT between difficulties

	0°	45°	90°	0–45°	0–90°
Accuracy(%)					
HC	89.6 (12.4)	79.6 (16.5)	72.2 (14.0)	−10.1 (9.5)	−17.5 (14.8)
PD	84.6 (15.2)	71.0 (18.1)	70.4 (17.0)	−13.8 (10.7)	−14.3 (12.4)
DLB	70.6 (22.2)^a^	59.4 (15.8)^a^	55.1 (10.8)^a^	−11.3 (17.1)	−15.6 (19.6)
RT (ms)					
HC	4896.99 (2256.98)	6250.27 (2722.86)	7176.14 (3186.59)	1353.28 (2167.24)	2279.15 (2150.84)
PD	4810.72 (1801.68)	5660.21 (2616.62)	5162.92 (2617.06)	848.76 (1530.98)	275.04 (1683.06)^b^
DLB	6202.17 (2904.05)	7130.58 (3985.40)	6040.89 (3178.23)	928.41 (2014.17)	−161.28 (1450.59)^b^

a = DLB < PD & HC, *P* < 0.001; b = < HC, *P* < 0.001.

Abbreviations: RT, Response time; HC, Healthy controls; PD, Parkinson's disease; DLB, Dementia with Lewy bodies.

### Neuropsychological Measures

#### Clinical Measures

Spearman correlations were performed within the DLB group across the three difficulty levels of the MR task (Table 3). No correlation was found between MMSE scores and MR performance across any of these difficulties. Negative correlations were found between the 90° and the PsycH‐Q (r_s_ (19) = −0.43, *P* = 0.05) and the 45° trials were trending towards significance (*P* = 0.06). Within the PsycH‐Q, the 45° difficulty was negatively correlated with the Hallucination and Psychosis subscale while the 90° difficulty was trending towards significance (r_s_ (19) = −0.43, *P* = 0.05, r_s_ (19) = −0.39, *P* = 0.07, respectively). Accuracy for each difficulty correlated with the Hallucination Phenotype scale (r_s_ (19) = −0.50, *P* = 0.02; r_s_ (19) = −0.45, *P* = 0.04; r_s_ (19) = −0.45, *P* = 0.04, respectively). Furthermore, each difficulty also had strong negative correlations with the visual hallucinations (VH) item of the MDS‐UPDRS scale (r_s_ (20) = −0.50, *p* = 0.02; r_s_ (19) = −0.69, *P* < 0.001; r_s_ (20) = −0.52, *P* = 0.01, respectively). Cognitive fluctuations as measured by the ODF were also negatively correlated with accuracy for the 0° and 45° difficulties (r_s_ (16) = −0.53, *P* = 0.02; r_s_ (16) = −0.51, *P* = 0.03, respectively) while the overall accuracy of the MR correlated with the ODF (r_s_ (16) = −0.56, *P* = 0.02).

**TABLE 3:** Group differences in cognitive measures

	HC	PD	DLB
Visuospatial			
Clock Drawing	10.00 (0.00)	9.68 (0.57)^a^	6.85 (3.15)^a,b^
RCF – Copy (z‐score)	0.40 (0.10)	0.34 (0.78)	−1.00 (2.08)^a^
RCF – Immediate (z‐score)	0.65 (0.65)	0.17 (0.95)	−1.10 (0.89)^a,b^
RCF – 20 min delay (z‐score)	0.74 (0.61)	0.09 (0.79)	−1.07 (1.03)^a,b^
Trails‐A (z‐score)	0.73 (0.72)	−0.07 (0.89)^a^	−3.17 (4.09)^a,b^
Executive Function^c^			
Backward Digit Span	7.35 (1.83)	7.18 (1.53)	4.77 (2.79)^a,b^
Verbal Fluency (animals) (z‐score)	0.79 (1.00)	0.61 (1.20)	−1.04 (1.10)^a,b^
Verbal Fluency (letter F) (z‐score)	0.60 (0.92)	0.56 (1.09)	−0.64 (1.03)^a,b^
Memory			
Digit span forward	10.35 (1.53)	11.36 (1.92)	9.10 (2.39)^b^
LM – Immediate Recall	12.10 (3.24)	9.59 (2.97)^a^	5.19 (3.28)^a,b^
LM – Delayed Recall	12.52 (2.20)	10.23 (2.88)^a^	6.40 (3.28)^a,b^

a = <HC; b = <DLB; c = Stroop and Trials B were excluded due to low completion numbers. *P* = 0.017.

Abbreviations: RCF, Rey Complex Figure; LM, Logical Memory.

### Cognitive Measures

Cognitive performance across the groups was compared (Table 3). As expected, the DLB group performed poorly with most measures. Dementia with Lewy body patients' performance on the MR task was correlated against measures of visuospatial, executive function and memory impairments (Table 4). Significant correlations indicated that the MR task was reliant on visuospatial and executive domains and conversely, there were no correlations between memory and MR performance.

**TABLE 4:** Clinical and cognitive spearman correlations with mental rotation accuracy

		Degrees of rotation (r_s_)		
	N	0°	45°	90°
Clinical				
MMSE	21	0.34	0.19	0.15
PsycH‐Q – Total	22	−0.39	−0.42	−0.43
Hallucinations and Psychosis	22	−0.26	**−0.43** ^**a**^	−0.35
Hallucination Phenotype	**22**	**−0.50** ^**a**^	**−0.45** ^**a**^	**−0.45** ^**a**^
VH – UPDRS	**22**	**−0.50** ^**b**^	**−0.69** ^**c**^	**−0.52** ^**b**^
One Day Fluctuations Scale	**18**	**−0.53** ^**a**^	**−0.51** ^**a**^	−0.29
MDSUPDRS Section III	22	0.16	−0.04	−0.12
Visuospatial				
Clock Drawing	**21**	0.46^a^	0.50^a^	0.55^a^
RCF – Copy	**15**	0.74^b^	0.79^b^	0.48
RCF – Immediate	15	0.38	0.44	0.51
RCF – 20 min delay	14	0.37	0.50	0.56^a^
Trails‐A	18	0.24	0.50^a^	0.54^a^
Executive Function^d^				
Backward Digit Span	**22**	0.47^a^	0.39	0.43^a^
Verbal Fluency (animals)	**21**	0.22	0.18	0.15
Verbal Fluency (letter F)	**21**	0.16	0.07	−0.15
Memory				
Digit span forward	21	0.27	0.33	0.20	
LM – Immediate Recall	21	0.29	0.10	0.04	
LM – Delayed Recall	20	0.34	0.03	0.05

a = *P* < 0.05; b = *P* < 0.01; c = *P* < 0.001; d = Stroop and Trials B were excluded due to low completion numbers.

Abbreviations: MMSE, Mini Mental State Examination; PsycH‐Q, Psychosis and Hallucinations Questionnaire; MDSUPDRS, Movement Disorder Society Unified Parkinson's Disease Rating Scale; VH, Visual Hallucinations; RCF, Rey Complex Figure; LM, Logical Memory.

## Discussion

The current study aimed to test the MR task as a potential tool to assist the differentiation of early DLB from patients with PD. In addition, it was hypothesized that the MR task may provide a sensitive measure of visuospatial impairment that could be used in symptomatic trials for such patients.

Despite being matched for age, education, and disease duration, DLB patients had significantly worse performance than PD patients on the MR task with poorer accuracy when the level of difficulty (degree of rotation) was increased. Similar patterns were seen between DLB patients and Controls.

Importantly, DLB patients were able understand and engage with the task across all levels of cognitive impairment. Performance accuracy was near chance for the 90° difficulty level, indicating that the limits of performance for DLB patients on the MR task lie between 0° and 90°. Moreover, the response times for DLB patients did not increase with the degrees of rotation, as observed in the HC group. This raises the question of whether the DLB group attempted to rotate the shapes, or if they used another technique with lower cognitive demand and less accuracy. Furthermore, if the DLB patients did not mentally rotate the shapes, although future studies would be required to determine the most sensitive range. With the current rotational degrees, the MR had an 80% accuracy of separating DLB from the other two groups. Additionally, with an accuracy threshold set to 80% the MR had high sensitivity, but low specificity. With the potential limit of the DLB ability at 90° of rotation, decreasing this rotation may increase the specificity without significantly decreasing the sensitivity.

As predicted MR performance in DLB was correlated with measures of visuospatial ability and executive function. For example, clock drawing was related to performance on the 45 and 90° conditions of the MR, which probably reflects the fact that patients would rely on internally visualizing the stimulus before successfully drawing a clock or comparing the shapes presented in the MR task.^42^ Working memory, as measured with backward digit span, also correlated with accuracy on the MR task. This is in line with previous research supporting the role of executive function in the manipulation of internally visualized stimuli.^43^ However, there was no difference in the cost of increasing the rotation between the groups. This was also supported with non‐significant interaction between groups and task difficulty. This could suggest that working memory is not a strong driver of the results. Alternatively, the lack of effect here could be due to the DLB group performing poorly at 45° and near chance for 90°, or low power due to a small sample size. Interestingly, deficits in memory as measured by recall and delayed recall were also not correlated with MR accuracy, suggesting a memory component may not be involved in the visualization and rotation of the object, or only have a minor role in the visualization and rotation of the object.

Within the DLB cohort, MR performance also appeared to be linked to visuoperceptive symptoms as patients who experienced more frequent and/or severe visual hallucinations were less accurate with the MR task. This is consistent with previous work that has linked poor performance on other visuospatial tests with presence of VH in DLB patients.^44^ In addition, a high occurrence of cognitive fluctuations was also associated with poor MR performance. The basis of this observation is less clear, as patients with severe fluctuations have also been shown to perform poorly on attentional tasks.^45^ Thus, it may be that patients with poor attention or concentration might struggle to perform MR.

The findings of this study suggest that DLB patients can complete the MR task when the degrees of rotation are not too great, given that their ability to visualize and manipulate objects is impaired but they were able to simply match stimuli at 0°. Thus, one advantage of the MR paradigm in future studies would be that the difficulty of the task may be manipulated and individualized to a patient's own performance. Thus, future symptomatic or disease modifying trials could optimize the sensitivity of the MR task to an individual patient's performance at baseline and then measure their response to an intervention ensuring that they were sensitive enough to measure and track effects. In addition, the MR task could be made increasingly difficult to probe subtle visuospatial impairment that might be present in proposed prodromal DLB cohorts.^13^ Indeed, in patients with isolated REM Sleep behavior disorder (a prodromal stage of Lewy body diseases), the MR task may be a useful tool to distinguish those patients who may phenoconvert to DLB instead of PD.^46^

Previous studies have demonstrated that non‐cognitively impaired individuals may employ strategies to assist them with mental rotation tasks. These strategies appear to improve accuracy and response time,^47^, ^48^ as well allowing participants to better adapt to changes in stimuli.^49^ In the current study, response times for DLB patients did not increase with the degrees of rotation, as observed in the HC group. This may indicate that the DLB group did not rotate the shapes to make their comparisons. Instead, they may have either employed a different strategy to compensate for their visuospatial impairment or they chose a random response. The former may have been used for the 45° condition, as the DLB patients scored well above chance. For the 90° condition however, the rotation may have been too high for their alternative strategy to be applied, resulting in random responses and the chance accuracy. While the current study did not focus on potential strategies that patients may have used during the MR task, it would be interesting to see if a cognitively impaired group would be able to employ such strategies and if these strategies would benefit their performance.

When interpreting the findings of this study there are several limitations that should be acknowledged. The first is that there was no comparison with another dementia cohort, such as PDD or AD. The objective of this study was to explore a task that could be helpful when patients are in the early stages of disease, when prognostication is probably most important, as well as to identify a task that might offer utility for the longitudinal tracking of patients and when assessing response to an intervention. Patients who transition from PD to PDD, will typically have similar neuropsychological profiles to those seen in DLB. Therefore, comparing DLB performance with AD would have been ideal given that the cognitive profiles of DLB and AD may be similar with many professionals misdiagnosing DLB patients with AD and vice versa. Interestingly, our findings did not identify any correlation between MR performance and memory impairment, which is the most prominently affected domain in patients with AD (especially early in the disease course) and so we would hypothesize that MR may be more sensitive in patients with DLB than AD. Additionally, few studies have looked at the sensitivity of visuospatial tests for differentiating between these two cohorts. Using the revised Addenbrooke's Cognitive Examination, which consist of memory measures as well as measures of executive function, attention and visuospatial ability, accuracy of differentiating between DLB and AD was vastly improved when emphasis was placed on the non‐amnestic scores.^50^ This highlights the utility of using non‐amnestic measures to differentiate between the two diseases. Indeed, Ota and colleagues^51^ found that using a battery of visuospatial tests had a high sensitivity, but low specificity at separating DLB from AD. This suggests that while visuospatial tests are very useful in differential diagnosis, more tasks may be needed to improve the overall specificity. Further research is needed to investigate differences in performance between DLB and AD patients, and if the diagnostic accuracy can be improved through adjustments to the degrees of rotation. The second potential limitation of this study was that the DLB patients were diagnosed with *probable* DLB, and this diagnosis was not confirmed by an autopsy. This creates the possibility that some of the DLB patients may have AD as a dominant pathology. Indeed, these AD dominant patients who had less severe fluctuations, VH and higher accuracy at the MR task may have been the driving force behind the correlations between MR performance and cognitive fluctuations and VH. If this were the case, this would further support the utility of the MR task as a means of separating DLB patients from similar pathologies such as AD. The third limitation of this study was that the majority (17/22) of the DLB patients were treated with a cholinesterase inhibitor to improve cognition.^52^ However, there was no difference in MR performance between those patients with and without AChEI treatment and one might anticipate that if anything, being on this therapy might have reduced our ability to detect an effect, which was not the case. The fourth limitation of the current study was its small sample size of DLB patients. However, this is a well‐phenotyped cohort of DLB patients diagnosed using the most recent criteria^7^ and the numbers tested were comparable with similar previous behavioral studies.^53^, ^54^, ^55^ Finally, it should be highlighted that many of the DLB patients tested were performing at chance for the 90° condition. This would have created a ceiling effect, thus reducing the amount of variance potentially leading to type II errors for these correlations. Future research would benefit from decreasing the maximum degrees of rotation, whilst also having more increments to identify the rotation where DLB patients begin to fail.

In summary, we identified that the MR task may be a sensitive tool for distinguishing DLB from early PD patients, with performance appearing to be correlated selectively with visuospatial, attention and working memory deficits but not long‐term memory. The ability of even quite severely impaired DLB patients to engage with this task suggests that its use should be explored in future clinical trials evaluating visuospatial impairment, where task difficulty could be matched to individual baseline performance.

## Author Roles

1. Research project: A. Conception, B. Organization, C. Execution; 2. Statistical Analysis: A. Design, B. Execution, C. Review and Critique; 3. Manuscript: A. Writing of the first draft, B. Review and Critique.

JRP: 1A, 1B, 1C, 2A, 2B, 3A

EM: 1B, 1C, 3B

KEM: 1B, 1C, 3B

AAM: 3B

GMH: 1A, 1B

SJL: 1A, 2C, 3B.

## Disclosures

**Ethical Compliance Statement:** Informed consent was provided by each patient and the study was approved by the Human Research Ethics Committee at Sydney University (HREC# 2012/2517). We confirm that we have read the Journal's position on issues involved in ethical publication and affirm that this work is consistent with those guidelines.

**Funding Sources and Conflicts of Interest:** The author(s) disclosed receipt of the following financial support for the research, authorship, and/or publication of this article: The work was supported by the Sydney Research Excellence Initiative 2020 grant, and ForeFront, a collaborative research group at the Brain and Mind Centre University of Sydney supported by NHMRC grants (#1037746 and #1095127). J.R.P. is a recipient of the Australian Postgraduate Award. E.M. is a recipient of scholarships from the National Health and Medical Research Council (NHMRC), the Australian and New Zealand Association of Neurologists (Gwen James Fellowship), and Avant Mutual.

(Advancement in Medicine Award). K.A.E.M. is supported by a Sydney University Fellowship. G.M.H. holds a NHMRC Senior Principal Research Fellow (#1079679). S.J.G.L. is supported by a NHMRC‐Australian Research Dementia Research Development fellowship. The author(s) declared no potential conflicts of interest with respect to the research, authorship, and/or publication of this article.

**Financial Disclosures for the Previous 12 Months:** The author(s) declared no potential conflicts of interest with respect to the research, authorship, and/or publication of this article.

## References

[1] Kane JPM , Surendranathan A , Bentley A , et al. Clinical prevalence of Lewy body dementia. Alzheimer's Res Ther 2018;10(1):19. 29448953 10.1186/s13195-018-0350-6 PMC5815202

[2] Aarsland D , Rongve A , Nore SP , et al. Frequency and case identification of dementia with Lewy bodies using the revised consensus criteria. Dement Geriatr Cogn Disord 2008;26(5):445–452. 18974647 10.1159/000165917

[3] Jellinger KA , Attems J . Prevalence and pathology of dementia with Lewy bodies in the oldest old: A comparison with other dementing disorders. Dement Geriatr Cogn Disord 2011;31(4):309–316. 21502762 10.1159/000327360

[4] Vann Jones SA , O'Brien JT . The prevalence and incidence of dementia with Lewy bodies: A systematic review of population and clinical studies. Psychol Med 2014;44(4):673–683. 23521899 10.1017/S0033291713000494

[5] Chertkow H , Feldman HH , Jacova C , Massoud F . Definitions of dementia and predementia states in Alzheimer's disease and vascular cognitive impairment: Consensus from the Canadian conference on diagnosis of dementia. Alzheimer's Res Ther 2013;5(Suppl 1):S2–S. 24565215 10.1186/alzrt198 PMC3981054

[6] Mueller C , Ballard C , Corbett A , Aarsland D . The prognosis of dementia with Lewy bodies. Lancet Neurol 2017;16(5):390–398. 28342649 10.1016/S1474-4422(17)30074-1

[7] McKeith IG , Boeve BF , Dickson DW , et al. Diagnosis and management of dementia with Lewy bodies: Fourth consensus report of the DLB consortium. Neurology 2017;89(1):88–100. 28592453 10.1212/WNL.0000000000004058 PMC5496518

[8] Foltynie T , Brayne CE , Robbins TW , Barker RA . The cognitive ability of an incident cohort of Parkinson's patients in the UK. The CamPaIGN study. Brain 2004;127(Pt 3):550–560. 14691062 10.1093/brain/awh067

[9] Nomura T , Inoue Y , Kagimura T , Nakashima K . Clinical significance of REM sleep behavior disorder in Parkinson's disease. Sleep Med 2013;14(2):131–135. 23218532 10.1016/j.sleep.2012.10.011

[10] McKeith IG , O'Brien J , Walker Z , et al. Sensitivity and specificity of dopamine transporter imaging with 123I‐FP‐CIT SPECT in dementia with Lewy bodies: A phase III, multicentre study. Lancet Neurol 2007;6(4):305–313. 17362834 10.1016/S1474-4422(07)70057-1

[11] Smirnov DS , Galasko D , Edland SD , Filoteo JV , Hansen LA , Salmon DP . Cognitive decline profiles differ in Parkinson disease dementia and dementia with Lewy bodies. Neurology 2020;94(20):e2076–e2087. 32332125 10.1212/WNL.0000000000009434 PMC7526670

[12] Kemp J , Philippi N , Phillipps C , et al. Cognitive profile in prodromal dementia with Lewy bodies. Alzheimer's Res Ther 2017;9(1):19. 28302161 10.1186/s13195-017-0242-1 PMC5356316

[13] McKeith IG , Ferman TJ , Thomas AJ , et al. Research criteria for the diagnosis of prodromal dementia with Lewy bodies. Neurology 2020;94(17):743–755. 32241955 10.1212/WNL.0000000000009323 PMC7274845

[14] Génier Marchand D , Postuma RB , Escudier F , de Roy J , Pelletier A , Montplaisir J , Gagnon JF . How does dementia with Lewy bodies start? Prodromal cognitive changes in REM sleep behavior disorder. Ann Neurol 2018;83(5):1016–1026. 29665124 10.1002/ana.25239

[15] Calderon J , Perry RJ , Erzinclioglu SW , Berrios GE , Dening TR , Hodges JR . Perception, attention, and working memory are disproportionately impaired in dementia with Lewy bodies compared with Alzheimer's disease. J Neurol Neurosurg Psychiatry 2001;70(2):157–164. 11160462 10.1136/jnnp.70.2.157 PMC1737215

[16] Quental N , Brucki S , Bueno O . Visuospatial function in early Alzheimer's disease—The use of the visual object and space perception (VOSP) battery. PloS one 2013;8(7):e68398. 23874610 10.1371/journal.pone.0068398 PMC3713013

[17] Warrington EK , James M . The Visual Object and Space Perception Battery. Bury St Edmunds, UK: Thames Valley Test Company; 1991.

[18] van de Beek M , van Steenoven I , van der Zande JJ , et al. Prodromal dementia with Lewy bodies: Clinical characterization and predictors of progression. Mov Disord 2020;35(5):859–867. 32048343 10.1002/mds.27997 PMC7317511

[19] Martini A , Weis L , Schifano R , et al. Differences in cognitive profiles between Lewy body and Parkinson's disease dementia. J Neural Transm (Vienna, Austria: 1996) 2020;127(3):323–330. 10.1007/s00702-019-02129-2 31898759

[20] Meyers J , Meyers K . Rey Complex Figure Test and Recognition Trial. Florida: Psychological Assessment Resources; 1995.

[21] Mondon K , Gochard A , Marque A , et al. Visual recognition memory differentiates dementia with Lewy bodies and Parkinson's disease dementia. J Neurol Neurosurg Psychiatry 2007;78(7):738–741. 17287240 10.1136/jnnp.2006.104257 PMC2117680

[22] Lee AC , Harris JP , Calvert JE . Impairments of mental rotation in Parkinson's disease. Neuropsychologia 1998;36(1):109–114. 9533393 10.1016/s0028-3932(97)00017-1

[23] Adduri CA , Marotta JJ . Mental rotation of faces in healthy aging and Alzheimer's disease. PloS one 2009;4(7):e6120. 19572013 10.1371/journal.pone.0006120 PMC2700266

[24] Bird CM , Chan D , Hartley T , Pijnenburg YA , Rossor MN , Burgess N . Topographical short‐term memory differentiates Alzheimer's disease from frontotemporal lobar degeneration. Hippocampus 2010;20(10):1154–1169. 19852032 10.1002/hipo.20715

[25] Flicker C , Ferris SH , Crook T , Reisberg B , Bartus RT . Equivalent spatial‐rotation deficits in normal aging and Alzheimer's disease. J Clin Exp Neuropsychol 1988;10(4):387–399. 3403702 10.1080/01688638808408247

[26] Lineweaver TT , Salmon DP , Bondi MW , Corey‐Bloom J . Differential effects of Alzheimer's disease and Huntington's disease on the performance of mental rotation. J Int Neuropsychol Soc 2005;11(1):30–39. 15686606 10.1017/S1355617705050034

[27] Oostra KM , Vereecke A , Jones K , Vanderstraeten G , Vingerhoets G . Motor imagery ability in patients with traumatic brain injury. Arch Phys Med Rehabil 2012;93(5):828–833. 22365480 10.1016/j.apmr.2011.11.018

[28] Shepard RN , Metzler J . Mental rotation of three‐dimensional objects. Science (New York, NY) 1971;171(3972):701–703. 10.1126/science.171.3972.701 5540314

[29] Bourrelier J , Kubicki A , Rouaud O , Crognier L , Mourey F . Mental rotation as an indicator of motor representation in patients with mild cognitive impairment. Front Aging Neurosci 2015;7:238. 26779010 10.3389/fnagi.2015.00238 PMC4688352

[30] Duncombe ME , Bradshaw JL , Iansek R , Phillips JG . Parkinsonian patients without dementia or depression do not suffer from bradyphrenia as indexed by performance in mental rotation tasks with and without advance information. Neuropsychologia 1994;32(11):1383–1396. 7877746 10.1016/0028-3932(94)00071-9

[31] Postuma RB , Berg D , Stern M , et al. MDS clinical diagnostic criteria for Parkinson's disease. Mov Disord 2015;30(12):1591–1601. 26474316 10.1002/mds.26424

[32] Psychology Software Tools I . E‐Prime 2.0. Pennsylvania, USA: Psychology Software Tools, Inc; 2013.

[33] Folstein MF , Folstein SE , McHugh PR . "Mini‐mental state". A practical method for grading the cognitive state of patients for the clinician. J Psychiatr Res 1975;12(3):189–198. 1202204 10.1016/0022-3956(75)90026-6

[34] Shine JM , Mills JMZ , Qiu J , et al. Validation of the psychosis and hallucinations questionnaire in non‐demented patients with Parkinson's disease. Mov Disord Clin Pract 2015;2(2):175–181. 30363832 10.1002/mdc3.12139 PMC6183006

[35] Shine JM , Muller AJ , O'Callaghan C , Hornberger M , Halliday GM , Lewis SJ . Abnormal connectivity between the default mode and the visual system underlies the manifestation of visual hallucinations in Parkinson's disease: A task‐based fMRI study. NPJ Parkinson's Dis 2015;1:15003. 28725679 10.1038/npjparkd.2015.3 PMC5516559

[36] Walker MP , Ayre GA , Cummings JL , Wesnes K , McKeith IG , O'Brien JT , Ballard CG . The clinician assessment of fluctuation and the one day fluctuation assessment scale: Two methods to assess fluctuating confusion in dementia. Br J Psychiatry 2000;177(3):252–256. 11040887 10.1192/bjp.177.3.252

[37] Reitan RM . Validity of the trail making test as an indicator of organic brain damage. Percept Mot Skills 1958;8(3):271–276.

[38] Wechsler D . Wechsler Adult Intelligence Scale‐Fourth Edition: Administration and Scoring Manual. San Antonio TX: NCS Pearson; 2008.

[39] Strauss E . A Compendium of Neuropsychological Tests: Administration, Norms, and Commentary. 3rd ed. New York: New York: Oxford University Press; 2006.

[40] Benton AL . Development of a multilingual aphasia battery. Progress and problems. J Neurol Sci 1969;9(1):39–48. 5820858 10.1016/0022-510x(69)90057-4

[41] Wechsler D . The Wechsler Memory Scale ‐ Fourth Edition (WMS‐IV). San Antonio, TX: Pearson Assessment; 2009.

[42] Pezzoli S , Cagnin A , Bussè C , Zorzi G , Fragiacomo F , Bandmann O , Venneri A . Cognitive correlates and baseline predictors of future development of visual hallucinations in dementia with Lewy bodies. Cortex 2021;142:74–83. 34217015 10.1016/j.cortex.2021.05.018

[43] Mazhari S , Moghadas TY . Abnormalities of mental rotation of hands associated with speed of information processing and executive function in chronic schizophrenic patients. Psychiatry Clin Neurosci 2014;68(6):410–417. 24920377 10.1111/pcn.12148

[44] Rosenblum Y , Bregman N , Giladi N , Mirelman A , Shiner T . Associations between visual hallucinations and impaired visuo‐spatial abilities in dementia with Lewy bodies. Neuropsychology 2021;35(3):276–284. 33970661 10.1037/neu0000728

[45] Phillips JR , Matar E , Martens KAE , Halliday GM , Moustafa AA , Lewis SJG . Evaluating the sustained attention response task to quantify cognitive fluctuations in dementia with Lewy bodies. J Geriatr Psychiatry Neurol 2019;891988719882093:333–339. 10.1177/0891988719882093 31672077

[46] Postuma RB , Iranzo A , Hu M , et al. Risk and predictors of dementia and parkinsonism in idiopathic REM sleep behaviour disorder: A multicentre study. Brain 2019;142(3):744–759. 30789229 10.1093/brain/awz030 PMC6391615

[47] Berneiser J , Jahn G , Grothe M , Lotze M . From visual to motor strategies: Training in mental rotation of hands. Neuroimage 2018;167:247–255. 27321046 10.1016/j.neuroimage.2016.06.014

[48] Nagashima I , Takeda K , Shimoda N , Harada Y , Mochizuki H . Variation in performance strategies of a hand mental rotation task on elderly. Front Hum Neurosci 2019;13:252. 31379545 10.3389/fnhum.2019.00252 PMC6659582

[49] Zhao B , Sala SD . Different representations and strategies in mental rotation. Q J Exp Psychol (Hove) 2018;71(7):1574–1583. 28856952 10.1080/17470218.2017.1342670

[50] Prats‐Sedano MA , Savulich G , Surendranathan A , et al. The revised Addenbrooke's cognitive examination can facilitate differentiation of dementia with Lewy bodies from Alzheimer's disease. Int J Geriatr Psychiatry 2021;36(6):831–838. 33275793 10.1002/gps.5483 PMC8247047

[51] Ota K , Murayama N , Kasanuki K , et al. Visuoperceptual assessments for differentiating dementia with Lewy bodies and Alzheimer's disease: Illusory contours and other neuropsychological examinations. Arch Clin Neuropsychol 2015;30(3):256–263. 25908613 10.1093/arclin/acv016

[52] Wesnes KA , McKeith IG , Ferrara R , et al. Effects of rivastigmine on cognitive function in dementia with lewy bodies: A randomised placebo‐controlled international study using the cognitive drug research computerised assessment system. Dement Geriatr Cogn Disord 2002;13(3):183–192. 11893841 10.1159/000048651

[53] Bliwise DL , Scullin MK , Trotti LM . Fluctuations in cognition and alertness vary independently in dementia with Lewy bodies. Mov Disord 2014;29(1):83–89. 24151106 10.1002/mds.25707 PMC3946909

[54] Johns EK , Phillips NA , Belleville S , et al. Executive functions in frontotemporal dementia and Lewy body dementia. Neuropsychology 2009;23(6):765–777. 19899835 10.1037/a0016792

[55] Park KW , Kim HS , Cheon SM , Cha JK , Kim SH , Kim JW . Dementia with Lewy bodies versus Alzheimer's disease and Parkinson's disease dementia: A comparison of cognitive profiles. J Clin Neurol 2011;7(1):19–24. 21519522 10.3988/jcn.2011.7.1.19 PMC3079155

